# The Role of ChatGPT and AI Chatbots in Optimizing Antibiotic Therapy: A Comprehensive Narrative Review

**DOI:** 10.3390/antibiotics14010060

**Published:** 2025-01-09

**Authors:** Ninel Iacobus Antonie, Gina Gheorghe, Vlad Alexandru Ionescu, Loredana-Crista Tiucă, Camelia Cristina Diaconu

**Affiliations:** 1 Faculty of Medicine, University of Medicine and Pharmacy Carol Davila Bucharest, 050474 Bucharest, Romania; ninel-iacobus.antonie@drd.umfcd.ro (N.I.A.); vladalexandru.ionescu92@gmail.com (V.A.I.); camelia.diaconu@umfcd.ro (C.C.D.); 2 Internal Medicine Department, Clinical Emergency Hospital of Bucharest, 105402 Bucharest, Romania; 3 Academy of Romanian Scientists, 050045 Bucharest, Romania

**Keywords:** antimicrobial resistance, artificial intelligence, AI chatbots, antibiotic stewardship, clinical decision support, ChatGPT

## Abstract

**Background/Objectives:** Antimicrobial resistance represents a growing global health crisis, demanding innovative approaches to improve antibiotic stewardship. Artificial intelligence (AI) chatbots based on large language models have shown potential as tools to support clinicians, especially non-specialists, in optimizing antibiotic therapy. This review aims to synthesize current evidence on the capabilities, limitations, and future directions for AI chatbots in enhancing antibiotic selection and patient outcomes. **Methods:** A narrative review was conducted by analyzing studies published in the last five years across databases such as PubMed, SCOPUS, Web of Science, and Google Scholar. The review focused on research discussing AI-based chatbots, antibiotic stewardship, and clinical decision support systems. Studies were evaluated for methodological soundness and significance, and the findings were synthesized narratively. **Results:** Current evidence highlights the ability of AI chatbots to assist in guideline-based antibiotic recommendations, improve medical education, and enhance clinical decision-making. Promising results include satisfactory accuracy in preliminary diagnostic and prescriptive tasks. However, challenges such as inconsistent handling of clinical nuances, susceptibility to unsafe advice, algorithmic biases, data privacy concerns, and limited clinical validation underscore the importance of human oversight and refinement. **Conclusions:** AI chatbots have the potential to complement antibiotic stewardship efforts by promoting appropriate antibiotic use and improving patient outcomes. Realizing this potential will require rigorous clinical trials, interdisciplinary collaboration, regulatory clarity, and tailored algorithmic improvements to ensure their safe and effective integration into clinical practice.

## 1. Introduction

Artificial intelligence (AI) systems based on large language models (LLMs) [1], such as OpenAI’s ChatGPT [2], Google’s Gemini [3], and Anthropic’s Claude [4], have become highly recognizable and popular due to their user-friendly interfaces and by offering natural, conversational interactions in the form of chatbots [5]. Their continuous advancements are rapidly transforming them into personal assistants, conveniently accessible through smartphones or other technological applications such as wearable devices, either via text or voice communication [6,7,8]. The adoption and usage of chatbots in the healthcare industry is expected to increase, particularly due to their potential to improve medical research processes, enhance access to medical information and provide personalized support [9,10,11,12]. Moreover, the decreasing supply of healthcare professionals is likely to accelerate this trend, as chatbots can help maximize the clinical efficiency of the remaining workforce by facilitating rapid access to information [13,14,15].

**Figure 1:**
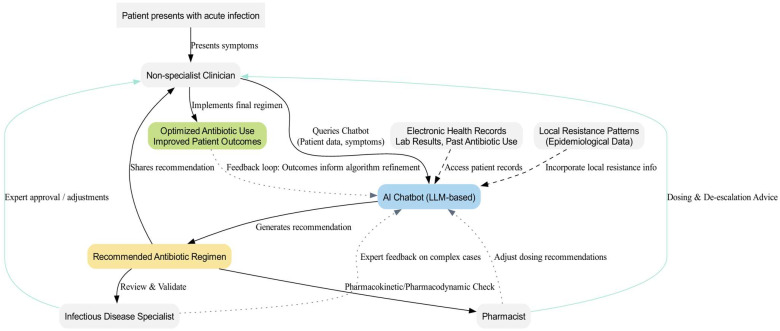
**Conceptual Framework of AI Chatbot Integration into Antibiotic Therapy Decision-Making.** This schematic illustrates how AI-driven chatbots, integrated with clinical data sources and supported by human expertise, can guide antibiotic prescribing. Starting from the patient’s presentation of acute infection, non-specialist clinicians access the chatbot to receive evidence-based recommendations derived from patient records, local resistance patterns, and established guidelines. Infectious disease specialists and pharmacists review the chatbot’s suggestions, providing necessary oversight to refine treatment plans and ensure adherence to stewardship principles. Feedback loops, including outcome monitoring and expert input, continuously inform algorithm refinement. In this way, AI chatbots serve as adjunct decision-support tools, working in tandem with human judgment to optimize antibiotic therapy and improve patient outcomes.

One of the most promising applications of LLMs is their ability to contribute to clinical decision support systems by recommending evidence-based antibiotic regimens. When implemented as chatbots in real-world scenarios, these systems could assist clinicians, reducing the rate of inappropriate antibiotic use [16,17,18,19,20] (Figure 1). Consequently, ongoing and future research may further substantiate their role in addressing the critical goal of combating antimicrobial resistance (AMR), a challenge that stands as one of the most pressing threats to global health. Current projections estimate that by 2050, even with the development of new antibiotics, annual deaths due to AMR could reach 10 million [21]. This underscores the urgency of developing innovative strategies to combat this growing threat.

Adapting to the evolving landscape of infectious diseases requires innovation. While humans develop new interventions, pathogens like *Escherichia coli*, *Staphylococcus aureus*, and *Klebsiella pneumoniae* exemplify the microbial adaptability that drives resistance mechanisms, perpetuating challenges for healthcare systems [22]. This dynamic outlines why new emergent and promising technological advancements should be approached. New and exciting research is already being published on this subject with very promising results, chatbots having the ability to structure medical notes and offer treatment suggestions while being easy to use and convenient [23,24,25].

In many clinical settings, patients presenting with acute infections are often managed by non-specialist physicians due to a shortage of infectious disease specialists. This situation frequently results in suboptimal antibiotic choices that do not fully adhere to established guidelines, potentially exacerbating antimicrobial resistance [26,27,28]. AI-driven chatbots could bridge the gap between complex clinical guidelines and everyday practice by providing non-specialist clinicians with timely, evidence-based treatment recommendations. These practical challenges in antibiotic stewardship have motivated this research into the role of AI chatbots in optimizing antibiotic therapy.

Thus, we pose a pivotal question: Can AI-driven chatbots play a meaningful role in optimizing antibiotic therapy? In this comprehensive review, we will explore this question by examining current research and practical applications to assess the potential impact of these technologies on antibiotic stewardship and clinical practice.

## 2. Materials and Methods

In conducting this comprehensive narrative review on the role of AI-based chatbots in optimizing antibiotic therapy, a systematic search strategy was employed to identify relevant literature across multiple scientific databases.

The following databases were utilized due to their extensive coverage of biomedical and technological research: PubMed; SCOPUS; Web of Science and Google Scholar, etc.

- Search Strategy

The literature search was carried out using a combination of keywords and Boolean operators to capture a broad range of studies related to AI chatbots and antibiotic therapy. The search queries were tailored to each database’s specific requirements, as outlined below:

The search strategy incorporated a combination of keywords related to:

1. AI Technologies: “chatbot*”, “conversational agent*”, “artificial intelligence”, “AI”, “ChatGPT”, “LLMs”, and names of specific AI systems (e.g., “Bard AI”, “Claude AI”).
2. Antibiotic Therapy: “antibiotic therapy”, “antibiotic prescribing”, “antimicrobial stewardship”, “antibiotic stewardship”, “antibiotherapy”.
3. Clinical Context: “error reduction”, “medication errors”, “prescribing errors”, “adherence”, “clinical decision support”, “decision-making”, “accessibility”, “education”, “patient education”, “health education”, “resource-limited settings”, “developing countries”.

Boolean operators such as “AND” and “OR” were used to combine these keywords effectively, allowing for a comprehensive search that included all relevant literature.

- Search Formulas used:

1. **SCOPUS**: TITLE-ABS-KEY(chatbot* OR “conversational agent*” OR “artificial intelligence” OR AI OR ChatGPT) AND TITLE-ABS-KEY(“antibiotic therapy” OR “antibiotic prescribing” OR “antimicrobial stewardship” OR “antibiotic stewardship”) AND TITLE-ABS-KEY(“error reduction” OR “medication errors” OR “prescribing errors” OR adherence OR “clinical decision support” OR “decision-making” OR accessibility OR education OR “patient education” OR “health education” OR “resource-limited settings” OR “developing countries”).
2. **Web of Science**: TS = (chatbot* OR “conversational agent*” OR “artificial intelligence” OR ChatGPT OR LLMs OR “Bard AI” OR “Claude AI” OR “Siri” OR “Alexa” OR “Google Assistant” OR “Microsoft Copilot” OR “Anthropic Claude” OR “IBM Watson” OR “Jasper AI” OR “Perplexity AI” OR “Replika”) AND TS = (“antibiotic therapy” OR “antibiotic prescribing” OR “antimicrobial stewardship” OR “antibiotic stewardship” OR “antibiotherapy”).
3. **PubMed**: (chatbot* OR “conversational agent*” OR “artificial intelligence” OR ChatGPT OR LLMs OR “Bard AI” OR “Claude AI” OR “Siri” OR “Alexa” OR “Google Assistant” OR “Microsoft Copilot” OR “Anthropic Claude” OR “IBM Watson” OR “Jasper AI” OR “Perplexity AI” OR “Replika”) AND (“antibiotic therapy” OR “antibiotic prescribing” OR “antimicrobial stewardship” OR “antibiotic stewardship” OR “antibiotherapy”).
4. **Google Scholar**: (“chatbot*” OR “conversational agent*” OR “artificial intelligence” OR “AI” OR “ChatGPT” OR “LLMs” OR “Bard AI” OR “Claude AI”) AND (“antibiotic therapy” OR “antibiotic prescribing” OR “antimicrobial stewardship” OR “antibiotic stewardship” OR “antibiotherapy”) AND (“error reduction” OR “medication errors” OR “prescribing errors” OR “clinical decision support” OR “patient education”).

- Inclusion and Exclusion Criteria

The selection of studies for inclusion in this review was guided by specific inclusion and exclusion criteria to ensure relevance and quality. We included articles published in English that were reviews, clinical studies, or original research articles focusing on artificial intelligence, chatbots, antibiotic therapy, antimicrobial stewardship, and related clinical decision support systems.

While no specific time restriction was set to capture both foundational and recent studies, emphasis was placed on literature published within the last five years to ensure relevance to current technologies.

Exclusion criteria comprised non-English publications, articles without accessible full texts, studies not directly related to the application of AI chatbots in antibiotic therapy or antimicrobial stewardship, and opinion pieces, editorials, and conference abstracts without accompanying full papers.

To enhance the comprehensiveness of this review, backward and forward citation tracking was employed; reference lists of included articles were examined to identify additional relevant studies; and citation databases were used to find newer articles citing the included studies.

- Rationale for Design and Data Synthesis

This review employs a narrative approach instead of a systematic review due to the heterogeneity of available literature and the emerging nature of AI chatbot technologies in antibiotic therapy. The existing research spans diverse disciplines, including computer science, clinical medicine, and public health, utilizing varied methodologies, study designs, and outcome measures, which complicates standardization under systematic review protocols. Additionally, many studies are descriptive or proof-of-concept, lacking the structured outcomes required for meta-analytical synthesis. A narrative synthesis was therefore chosen as the most appropriate method to integrate findings, identify trends, address challenges, and suggest future directions. Although a formal quality appraisal was not performed, the included studies were evaluated for methodological soundness and relevance to the research question, with key information extracted on study design, sample size, AI chatbot characteristics, clinical applications, outcomes, benefits, challenges, and limitations. The narrative synthesis approach enabled a comprehensive overview of the current evidence, balancing both established knowledge and emerging developments in this rapidly evolving field.

## 3. Current Trends in Antimicrobial Resistance: Recent Data and the Need for Innovative Solutions

**Figure 2:**
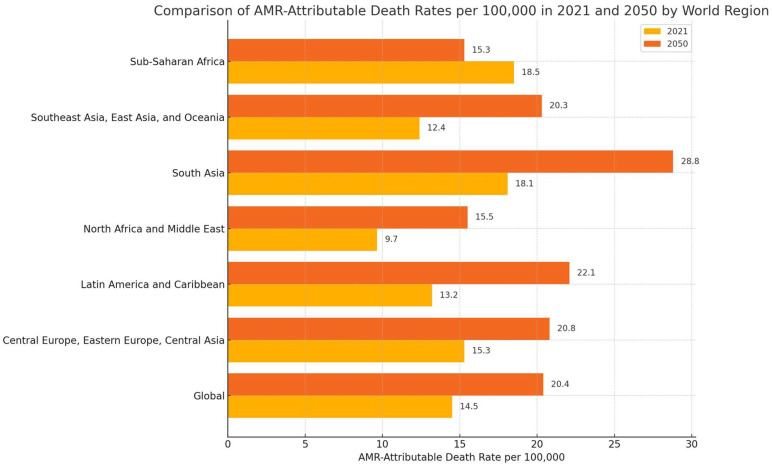
**Comparison of AMR-Attributable Death Rates per 100,000 in 2021 and 2050 by region.** This figure compares AMR-attributable death rates per 100,000 population across global regions for the years 2021 (yellow) and 2050 (orange), highlighting significant regional disparities and projected trends The data was adapted from [21].

Recent analyses underscore the devastating global impact of antimicrobial resistance (AMR). Significant disparities in AMR-related mortality across regions highlight the need for tailored, region-specific strategies. Globally, the AMR-attributable death rate was approximately 14.5 per 100,000 in 2021, with projections suggesting an increase to 20.4 by 2050. When comparing world regions, Central Europe, Eastern Europe, and Central Asia reported an AMR-attributable death rate of around 15.3 per 100,000 in 2021, with an anticipated rise to 20.8 by 2050. In contrast, South Asia exhibited a notably higher rate of 18.1 per 100,000 in 2021, with projections indicating a significant increase to 28.8 by 2050 [21] (Figure 2).

When considering specific pathogens, in the European region, a systematic review focusing on drug-resistant bloodstream infections (BSIs) identified alarmingly high mortality odds ratios for pathogens like carbapenem-resistant *Klebsiella pneumoniae* and vancomycin-resistant enterococci, underscoring the need for targeted, pathogen-specific interventions [29]. Meanwhile, the European Centre for Disease Prevention and Control (ECDC) reports a mixed picture: MRSA rates are declining, yet carbapenem-resistant *Klebsiella pneumoniae* are on the rise and surpassing reduction targets set for 2030 [30]. Data from the World Health Organization’s (WHO) Global Antimicrobial Resistance and Use Surveillance System (GLASS) add more complexity, revealing critical gaps in testing and infrastructure—especially in low- and middle-income countries—making it clear that what works in one region may not apply in another [31].

The COVID-19 pandemic has further complicated this landscape. Some studies found no overall surge in Gram-positive resistance, yet subtle upticks in Gram-negative resistance emerged in places lacking robust prevention measures [32]. In intensive care units (ICUs), the challenge is even greater: timely, broad-spectrum antibiotics are often necessary to save lives, but without careful de-escalation and pharmacokinetic-pharmacodynamic optimization, such strategies risk fueling AMR [33]. Pediatric care faces its own hurdles. Many community hospitals lack pediatric-specific data and expertise, forcing them to rely on evidence-based principles and stewardship frameworks that can reduce adverse events like *Clostridioides difficile* infections and improve safety [34,35].

Geographical disparity plays a pivotal role. In Asia, antimicrobial stewardship (AMS) programs must adapt to resource constraints, limited microbiological data, and varying levels of staff awareness to effectively curb AMR [36]. Elsewhere, low- and middle-income settings grapple with self-medication, poor infrastructure, and rampant suboptimal prescribing, yet success stories exist. By starting with modest goals—like cutting carbapenem use or developing locally relevant guidelines—and building toward more complex interventions, even under-resourced hospitals can make progress [36,37]. The outpatient realm requires equally careful tactics: strategies like the “Five Ds” (right diagnosis, drug, dose, duration, and de-escalation) in managing urinary tract infections can trim down unnecessary prescriptions [38]. Better diagnostics, like reflex urine cultures or modified reporting, help distinguish symptomatic infections from asymptomatic bacteriuria, guiding more appropriate antibiotic use.

This intricate web of AMR challenges extends to critical conditions such as multidrug-resistant sepsis. Here, the stakes are life-and-death, and standard approaches struggle against organisms like carbapenem-resistant Enterobacteriaceae [39]. Advanced diagnostics, coupled with real-time surveillance and stewardship, become indispensable. Improving patient outcomes, reducing healthcare costs, and trimming hospital stays hinge on early, targeted therapy that anticipates resistance patterns. Traditional methods alone cannot keep pace with these evolving threats. Instead, what emerges is a call for innovative tools, including artificial intelligence and personalized treatments, as well as novel preventive measures like vaccines and monoclonal antibodies [29].

In the end, these findings [29,30,31,32,33,34,35,36,37,38,39] converge on a single truth: AMR is not just a clinical or microbiological issue; it is a multifaceted global health emergency that demands adaptive, data-driven, and context-specific solutions. Established stewardship principles remain fundamental, but accelerating trends and mounting complexity mean we must also embrace novel diagnostics, interdisciplinary collaboration, and advanced decision-support tools. Artificial intelligence and other cutting-edge interventions hold the promise of integrating diverse data streams—from local resistance patterns to patient history—into coherent, actionable recommendations. Achieving this synergy is challenging, yet essential. This is the path forward if we aim to outrun the evolution of drug-resistant pathogens and restore a semblance of control over the use of our most precious therapeutic resources.

These emerging trends in antimicrobial resistance, coupled with the heterogeneous success of current interventions, highlight a pressing need for advanced, adaptable solutions. It is within this context that AI-driven chatbots—capable of rapidly integrating diverse data streams and providing near-real-time recommendations—may offer a strategic advantage. In the sections that follow, we will examine how these systems function, where they excel, and what obstacles must be overcome to realize their full potential in optimizing antibiotic therapy.

## 4. AI-Based Chatbots: From Design Principles to Practical Applications

### 4.1. What Are AI-Based Chatbots?

AI-based chatbots, such as ChatGPT, Gemini, and Claude, are complex systems designed to simulate human dialogue. Building on foundational research in conversational artificial intelligence dating back to 1966, these systems demonstrate significant potential as continuous advancements enhance their capabilities and broaden their applications [40,41,42]. These chatbots operate on the principle of processing input information and generating corresponding output [43]. To function effectively, they rely on large language models, such as GPT (Generative Pre-trained Transformer), which use deep learning algorithms trained on immense datasets containing billions of words [44,45,46]. This training enables them to predict relevant information and generate contextually appropriate responses based on the input they receive. Specifically designed for conversational tasks, these systems excel at answering queries and assisting with a wide range of activities [47,48].

### 4.2. How Do These Models Work?

By leveraging the transformer architecture introduced by Vaswani et al., AI systems utilize self-attention mechanisms to weigh the importance of each word in a sequence. In practical terms, this means while chatbots can parse lengthy clinical notes, their lack of actual clinical reasoning may cause them to miss subtle safety cues, reinforcing the importance of complementary human expertise. This allows the model to effectively capture contextual relationships, enabling it to understand linguistic nuances and dependencies over long text spans [49].

Before becoming operational, transformer-based AI systems undergo a critical step known as pre-training. During this phase, the model is exposed to massive amounts of data, allowing it to learn grammar, semantics, and general world knowledge. Following pre-training, these systems undergo fine-tuning, which adapts them for specific applications, such as chatbots (conversational agents). This phase often employs reinforcement learning with human feedback (RLHF) to align the system’s responses with user expectations and ensure higher-quality outputs [5,50].

These AI systems process inputs as tokenized text sequences, where tokens represent fragments of data such as words. These tokens are embedded into high-dimensional vectors, which are then processed iteratively by the transformer’s algorithm. Through multiple layers of self-attention and feed-forward networks, the model generates contextually coherent responses one token at a time, building outputs until the sequence is complete [49,51].

### 4.3. Capabilities and Limitations of AI-Based Chatbots

Transformer-based AI models, such as GPT, are particularly adept at generating coherent and contextually appropriate text. They excel in tasks like summarizing information, translating languages, and answering questions. These capabilities are made possible by their ability to model long-range dependencies and capture nuanced linguistic structures effectively [5,51,52,53,54].

However, despite their impressive performance, these models have notable limitations. They lack intrinsic understanding or reasoning, functioning as statistical systems that generate predictions based on patterns in their training data. As a result, any flaws, biases, or limitations in the training data are inherently carried over into the model’s outputs. These systems cannot recognize or correct errors in the information they produce [43,50].

A major issue with transformer-based AI systems is their susceptibility to hallucinations—outputs that lack a basis in the input data or reality. These “hallucinations” occur when the model generates plausible-sounding but factually incorrect or fictional information. Such outputs can undermine trust in the system and pose challenges, particularly in high-stakes applications like medicine or legal analysis [55,56,57].

Understanding these underlying computational principles is not merely technical background; it reveals why chatbots can generate relevant, context-aware suggestions yet still struggle with complex clinical reasoning. For example, while self-attention mechanisms enable the model to parse long clinical notes and flag potential antibiotic choices, the absence of true clinical understanding explains why the system may overlook critical safety cues or fail to adjust therapy as patient conditions evolve.

### 4.4. Practical Applications of AI-Based Chatbots in Healthcare

Beyond their theoretical application in optimizing antimicrobial therapy, large language models implemented as chatbots demonstrate broader potential that will be discussed briefly in this section. Thus, despite being in the early stages of development, tools like ChatGPT are already being explored for practical uses, including supporting clinical decision-making, improving medical education, and minimizing errors.

AI-based decision support systems capable of processing dynamic text data in real-time offer significant potential. By evaluating incoming information as it is received, these systems ensure decision-makers have access to the most current and relevant data. Additionally, their ability to integrate diverse dynamic text sources enables a comprehensive analysis of information from multiple channels. Leveraging advanced algorithms, these systems can effectively interpret data to support well-informed decision-making [58,59,60,61].

In a systematic review by Frangoudes et al. (2021), chatbots functioning as virtual patients were shown to play a valuable role in medical education. These systems provide real-time feedback during interactions, allowing medical students to improve their clinical reasoning and communication skills. The review highlights that virtual patient chatbots have been used to simulate realistic patient encounters, enabling students to practice history-taking, diagnostic reasoning, and empathy in a controlled environment. Notably, studies included in the review emphasize the benefits of automatic feedback modules, which help students refine their skills through repeated scenarios and diverse case simulations. However, the review also notes the limitations of chatbot systems, including their reliance on predefined question-answer patterns and challenges in generating highly naturalistic dialogue, which may hinder deeper learning experiences for advanced users [61].

When comparing the performance of AI chatbots, (ChatGPT-4o and Claude-3), against Family Medicine residents, findings published by Huang et al. (2024) suggest that, although AI chatbots can process vast amounts of medical information and provide consistent responses, their current capabilities in reducing diagnostic errors are limited. The prevalence of logical errors highlights the need for further refinement in their reasoning algorithms. Therefore, while AI chatbots hold potential as supplementary tools in medical education and practice, they should not replace human judgment, especially in complex cases involving diagnostic uncertainty [62].

In their narrative review, Abavisani et al. examine the potential of AI-driven chatbots in addressing antibiotic resistance, highlighting their capacity to enhance clinical workflows [16]. These AI systems integrate seamlessly with electronic health records, offering patient-specific antibiotic recommendations and supporting antimicrobial stewardship by reducing inappropriate prescriptions. Furthermore, they promote evidence-based decision-making and improve adherence to clinical guidelines. Despite these advancements, significant gaps remain. The review lacks detailed discussion on the cost-effectiveness of chatbots in resource-limited settings, cultural barriers to adoption, and their integration within multidisciplinary care teams. While emphasizing the need to address biased or incomplete training data and the inability to replicate human clinicians’ nuanced reasoning, the review provides limited insight into pilot programs or real-world implementation strategies.

Building on these findings, this review seeks to expand the discussion by incorporating underexplored areas such as algorithmic refinements tailored to diverse healthcare settings and novel models designed to mitigate resistance patterns in localized scenarios. Additionally, the potential of chatbots in facilitating collaborative care, particularly in team-based settings, and their use in remote areas with limited access to specialists is explored. By addressing these gaps and contextualizing AI advancements within specific case studies, this comprehensive review contributes to the advancement of our current understanding of the practical application of AI chatbots in antibiotic therapy.

## 5. The Use of AI-Based Chatbots in Antibiotic Therapy

While AI-based chatbots have shown promise in various aspects of healthcare, their application in antibiotic therapy remains relatively underexplored. A comprehensive search of relevant databases yielded only four [17,18,19,20] (Table 1) experimental studies directly evaluating the use of chatbots in this domain. This paucity of research highlights a significant gap in the literature and underscores the urgent need to investigate how AI-driven chatbots can optimize antibiotic use. In the following section, we will delve into these studies, examining their findings and implications for clinical practice and future research.

Maillard et al. (2024) evaluate the role of ChatGPT-4 as a decision-support tool in managing bloodstream infections (BSIs), comparing its recommendations with those of infectious disease consultants [20]. Conducted in a tertiary care hospital, this prospective study analyzed 44 cases of BSIs, focusing on diagnostic accuracy, treatment planning, and follow-up care. While ChatGPT-4 provided satisfactory diagnostic workups in 80% of cases, its antibiotic therapy recommendations were often suboptimal, with harmful suggestions in up to 16% of cases. The study highlights the chatbot’s potential in generating structured management plans but underscores significant safety concerns, particularly for severe infections. Key limitations include a single-center design, reliance on standardized prompts, and challenges with ambiguous clinical data. Despite these limitations, the findings demonstrate the need for further refinement and integration of chatbots into clinical workflows, emphasizing their role as supplementary tools under expert supervision. Future research should focus on multicenter validation, improved dataset diversity, and hybrid models combining AI outputs with expert oversight [20].

The study by De Vito et al. (2024) evaluates the theoretical knowledge and prescriptive accuracy of ChatGPT-4 in managing bacterial infections compared to infectious disease residents and specialists [19]. The researchers assessed 72 questions across four domains: true/false queries, open-ended questions, and clinical cases with antibiograms related to endocarditis, bloodstream infections, pneumonia, and intra-abdominal infections. The questions, designed with varying difficulty levels, were reviewed by blinded experts for accuracy, completeness, and clinical relevance. Key findings revealed that ChatGPT-4 and its trained version performed comparably to human participants in theoretical questions, with correct answers in approximately 70% of cases. For open-ended questions, ChatGPT-4 demonstrated higher accuracy and completeness than residents and specialists, particularly when using the trained model. However, in clinical case management, ChatGPT-4 struggled with interpreting antibiograms and often recommended outdated treatments, such as colistin over newer options. Both ChatGPT-4 versions exhibited a tendency to overtreat and recommend unnecessarily long treatment durations. The study highlights the potential of AI tools like ChatGPT in enhancing medical education and providing preliminary diagnostic insights. However, limitations such as reliance on hypothetical data, single-center design, and lack of nuanced clinical reasoning underscore the necessity of human oversight. Future research should focus on refining AI algorithms for real-world applications, including expanding training datasets and validating these tools in multicenter trials. This study underscores the complementary role of AI in healthcare, particularly for education and support, while reaffirming the irreplaceable value of expert clinical judgment [19].

In a study conducted by Sarink et al. (2023), the capabilities and limitations of ChatGPT version 3.5 were examined, more specifically in providing antimicrobial recommendations for real-world infection scenarios [18]. The researchers evaluated ChatGPT’s responses based on criteria such as appropriateness, safety, consistency, and adherence to antimicrobial stewardship principles. The findings revealed that ChatGPT demonstrated an ability to understand and summarize clinical scenarios effectively when provided with explicit details. The model generated coherent, grammatically sound responses that often included disclaimers recommending consultation with a specialist. However, critical limitations were identified. ChatGPT frequently failed to distinguish between important and unimportant clinical factors, showed inconsistency when re-asked similar questions, and exhibited “failure modes” that led to the repeated provision of unsafe advice. Notably, the model often overlooked clinical safety cues and nuanced considerations such as the duration of therapy and the implications of source control. The authors highlighted that while ChatGPT possesses sufficient training data to generate plausible recommendations, its lack of situational awareness and inferential reasoning poses significant barriers to safe clinical implementation. These deficits underscore the model’s unreliability in complex medical decision-making, as it frequently misinterpreted scenarios of increasing complexity. To address these issues, the study proposed a qualitative assessment framework aimed at guiding future safety evaluations of AI systems across medical specialties. The strengths of the study include its focus on practical clinical scenarios and the systematic analysis of ChatGPT’s performance. Limitations involve the focus on non-chronic cases, variability in the information provided to ChatGPT, and differences in clinician-written clinical scenarios, which could influence reproducibility despite a high inter-reader reliability rate. The findings align with broader concerns regarding AI’s readiness for unsupervised application in medicine, contrasting with studies that show greater AI reliability in structured tasks like medical examination questions. The authors emphasize that future research should focus on refining AI systems to improve situational awareness and integrating interdisciplinary expertise to ensure safe and effective use in clinical practice. Given the rapid evolution of generative AI, understanding its implications for patient care is of urgent importance [18].

A study by Howard et al. (2023) evaluated ChatGPT’s performance in providing antimicrobial advice using eight hypothetical infection scenario-based questions [17]. The responses were assessed across key parameters, including appropriateness, consistency, safety, and adherence to antimicrobial stewardship principles, culminating in the development of an LLM medical safety assessment framework. The authors highlighted ChatGPT’s ability to recognize natural language, generate coherent and accurately summarized responses, and offer management options accompanied by disclaimers outlining the limitations of its recommendations. However, significant limitations were identified, such as the inability to differentiate between important and less relevant clinical factors and frequent omissions of critical considerations in increasingly complex scenarios. While the regimens proposed by ChatGPT were generally appropriate for the diagnoses and demonstrated correct antimicrobial spectrum selection, therapy duration recommendations varied inconsistently. ChatGPT often assumed that antimicrobial choice was the primary issue, potentially reflecting biases in the presented queries. Additionally, its handling of contraindications was inconsistent, and it sometimes entered “failure modes,” providing unsafe advice despite repeated corrections. Most responses included disclaimers advising consultation with infection specialists, but the lack of situational awareness, inference capabilities, and consistency were identified as barriers to clinical implementation. The study underscores the potential of ChatGPT as a decision-support tool while emphasizing the need for further refinement, particularly in managing complex clinical scenarios, and suggests that interdisciplinary expertise will be essential for its safe integration into medical practice [17].

These studies indicate that while chatbots can offer structured, evidence-based recommendations, human expertise remains indispensable. Infectious disease specialists, pharmacists, and general clinicians must remain vigilant, reviewing and interpreting chatbot outputs, particularly in complex or uncertain scenarios. The integration of AI should thus be seen as a collaborative partnership rather than a replacement—human judgment ensures that data-driven suggestions align with patient-specific nuances and uphold the highest standards of care.

## 6. Benefits of Chatbots in Optimizing Antibiotic Therapy

The integration of AI-driven systems, such as ChatGPT, into antimicrobial stewardship programs demonstrates potential in addressing key challenges in antibiotic prescribing. Studies by Maillard et al. (2024) and De Vito et al. (2024) emphasize ChatGPT’s ability to provide structured recommendations and preliminary diagnostic insights, showcasing its potential to reduce errors in prescribing through real-time decision support and guideline adherence [19,20]. However, significant limitations, including inconsistent handling of clinical nuances and unsafe recommendations in complex cases, highlight the need for human oversight and algorithmic refinement. Research by Sarink et al. (2023) further identifies ChatGPT’s struggles with situational awareness and inferential reasoning, which are critical for distinguishing between clinically relevant factors, ensuring adherence to antibiotic stewardship program principles, and tailoring recommendations to specific scenarios [18]. While Howard et al. (2023) illustrate ChatGPT’s competency in generating coherent responses and addressing antimicrobial spectrum selection, its inconsistency in therapy duration recommendations and handling of contraindications underlines the challenges of achieving reliable clinical implementation [17]. Collectively, these studies underscore the utility of AI in supporting rapid decision-making, especially in emergencies, and its promise in resource-limited settings by providing accessible diagnostic support. Additionally, its ability to enhance education for both clinicians and patients on responsible antibiotic use demonstrates its value as a complementary tool in antimicrobial stewardship efforts, albeit with the necessity of expert supervision to ensure safety and efficacy.

## 7. Beyond Chatbots: Other AI Applications in Optimizing Antibiotic Therapy

Given the extensive and rapidly growing body of research on the potential applications of AI systems in optimizing antibiotic therapy—where chatbots have only recently gained prominence—we feel it is important to briefly mention other AI applications to highlight the importance of new research being conducted in this exciting field.

Machine learning algorithms are being integrated with clinical microbiology to streamline diagnostic workflows. For instance, AI-based systems incorporating mass spectrometry data and machine learning can expedite the detection of multidrug-resistant pathogens, significantly reducing turnaround times for diagnostic decisions while improving treatment outcomes [63,64,65]. These tools enhance antimicrobial stewardship by predicting resistance patterns and optimizing antibiotic selection, especially in critical care settings where timely decisions are paramount [66,67].

In pediatric medicine, AI models are particularly useful for combating antimicrobial resistance. They support tailored antimicrobial stewardship strategies, identifying resistance trends and guiding appropriate therapy [34,68,69]. Predictive analytics driven by AI also allow for individualized treatment recommendations, factoring in patient-specific data like comorbidities, demographics, and infection history. This precision facilitates better empiric antibiotic choices, minimizing overprescription and the selection of broad-spectrum antibiotics [64,67].

Furthermore, AI-driven platforms are integrating real-time data from electronic health records to support empirical therapy decisions, leveraging local epidemiological trends to refine antimicrobial stewardship practices. These platforms contribute to early detection and intervention, which are crucial in managing infections in resource-limited settings [66,67,70]. However, barriers remain, including the need for extensive validation, regulatory frameworks, and equitable access to these technologies to ensure their effectiveness across diverse clinical contexts [63,68]. This comprehensive view of AI applications underscores its transformative potential while calling for multidisciplinary collaboration to address implementation challenges.

## 8. Challenges and Limitations

Algorithmic bias, data confidentiality concerns, and insufficient clinical validation are critical barriers to the effective integration of AI-based chatbots like ChatGPT in optimizing antibiotic therapy. Algorithmic bias arises from non-representative training datasets that often fail to account for diverse patient populations or prioritize high-resource settings, resulting in recommendations poorly suited for underrepresented groups or low-resource environments. Even with unbiased data, structural limitations in algorithms can amplify disparities, leading to skewed outputs that undermine trust among clinicians and patients. These challenges are exacerbated by the opacity of many AI systems, which operate as “black boxes”, making it difficult to trace and rectify biases. Mitigating these issues requires robust validation methods, diverse datasets, and interdisciplinary oversight to ensure equitable AI implementation [71,72,73].

**Figure 3:**
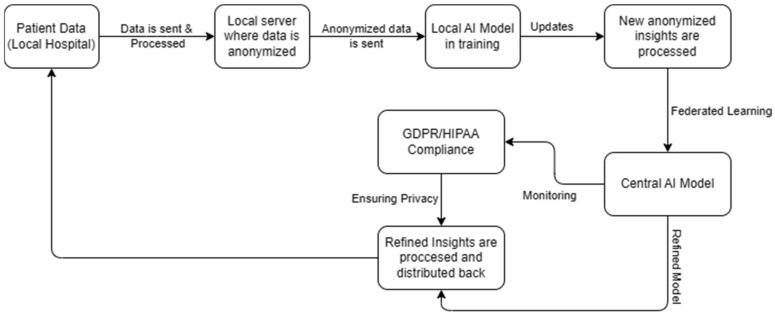
**Privacy and Compliance Measures in Federated Learning for AI Models.** The data flow diagram illustrates how patient data is securely processed and integrated into an AI model using federated learning, ensuring compliance with privacy regulations like GDPR and HIPAA. It highlights each step in the data pipeline, ensuring transparency and accountability in data handling.

Equally important, patient data confidentiality presents another significant concern, as compliance with regulations like the General Data Protection Regulation (GDPR) and the Health Insurance Portability and Accountability Act (HIPAA) is paramount [74,75,76]. AI systems risk privacy breaches through improper anonymization or storage of sensitive information for fine-tuning, eroding user trust. Addressing these challenges requires transparent data handling, adherence to international privacy standards, and explainable AI frameworks to safeguard sensitive information (Figure 3) [77].

The integration of AI-based chatbots like ChatGPT in healthcare, particularly in optimizing antibiotic therapy, faces several legal, technical, and institutional challenges that demand attention. Responsibility for errors arising from AI use remains contentious; under the EU Medical Device Regulation (MDR) and In Vitro Diagnostic Regulation (IVDR), manufacturers are primarily accountable for the safety and intended use of AI-based medical devices. However, healthcare providers deploying these tools share liability, especially when AI recommendations are applied beyond approved operational boundaries, highlighting the need for legal clarity on shared accountability between developers and clinicians. Regulatory frameworks, including the MDR, IVDR, GDPR in Europe, and the FDA’s lifecycle approach in the U.S., underscore the necessity for rigorous testing, certification, and post-market surveillance of AI systems, ensuring their safe and effective deployment. These regulations also demand transparency and adherence to pre-approved algorithm change protocols, particularly for adaptive AI systems that evolve during real-world use [78,79,80,81,82].

Compounding these challenges is the lack of extensive clinical validation and large-scale studies to confirm the efficacy and safety of chatbots in real-world settings. Many existing studies rely on hypothetical data, failing to capture clinical complexities, which hinders the establishment of benchmarks for evaluating AI performance. Without comprehensive validation, reliance on these systems risks perpetuating errors, particularly in high-stakes contexts such as antibiotic stewardship. Addressing these gaps demands rigorous multicenter trials and interdisciplinary collaboration to ensure these tools transition from experimental technologies to reliable components of healthcare delivery [29,35].

Encouragingly, pilot programs in some institutions are testing frameworks for unbiased dataset curation, including the use of representative patient cohorts and continuous model retraining against diverse clinical data. Similarly, privacy-preserving techniques, such as federated learning and differential privacy, allow models to improve without centralizing sensitive patient information [59,83,84,85,86,87]. Early collaborations between AI developers, ethicists, and legal experts are beginning to produce guidance documents and standardized operating procedures, helping clarify liability issues and ensure compliance with regulatory frameworks like the MDR, IVDR, and FDA’s lifecycle approach. Such practical steps illustrate that while the challenges are substantial, they are not insurmountable.

### Proposed Strategies to Address AI Chatbot Limitations

Building on our earlier discussion of key limitations, we suggest a series of targeted measures—ranging from diverse training datasets and human oversight in high-stakes cases to refined prompt-engineering and privacy-preserving techniques—that together can enhance chatbot reliability, reduce algorithmic bias, prevent unsafe recommendations, minimize hallucinations, and safeguard patient data.

- **Algorithmic Bias.** Training datasets that underrepresent certain patient populations can produce skewed recommendations and exacerbate health disparities. To reduce bias, AI chatbots need the following:
  ▪ Dataset Expansion: Collaborations across diverse institutions to include various demographics and clinical contexts.
  ▪ Regular Testing: Frequent evaluations with representative patient cohorts.
  ▪ Feedback Loops: Clinicians and pharmacists flag questionable outputs, prompting updates to training processes.
- **Unsafe Advice/Missed Clinical Nuances.** Chatbots can overlook key patient factors or propose outdated therapies, underscoring the need for human oversight. Suggested fixes are as follows:
  ▪ Safety Checks: Automated alerts for allergies, interactions, or guideline mismatches.
  ▪ Specialist Review: Infectious disease experts or pharmacists approve final suggestions, especially in high-stakes scenarios.
  ▪ Contextual Prompts: Structured reminders for comorbidities, patient age, and recent antibiotic history.
- **Hallucinations and Misinformation.** When chatbots confidently provide incorrect information, major clinical risks arise. Mitigation approaches include the following:
  ▪ Model Refinement: Carefully crafted prompts or limiting response scope.
  ▪ Step-by-Step Reasoning: Documenting the model’s reasoning to spot errors.
  ▪ Validation Layers: Cross-checking outputs against trusted sources (antibiograms, guidelines).
- **Data Privacy and Confidentiality.** Compliance with regulations like GDPR and HIPAA is essential. Protective methods include the following:
  ▪ Federated Learning: Training models locally at each institution without centralizing sensitive data.
  ▪ Differential Privacy: Introducing controlled “noise” to prevent re-identification.
  ▪ Secure Enclaves: Using encrypted, access-controlled environments for AI model tuning.

By adopting these solutions—ranging from diverse data and specialist oversight to refined prompt-engineering and secure data handling—AI chatbots can become safer, more accurate, and more trusted aids in clinical practice.

## 9. Conclusions

This comprehensive narrative review reveals that AI-based chatbots, particularly ChatGPT, hold considerable promise in optimizing antibiotic therapy. By assisting with evidence-based antibiotic selection, offering structured treatment recommendations, supporting preliminary diagnostics, and providing educational guidance, these systems can enhance adherence to clinical guidelines and strengthen antimicrobial stewardship. When appropriately integrated, chatbots may help non-specialist clinicians make more informed decisions and improve patient outcomes, ultimately contributing to global efforts against antimicrobial resistance.

Despite these encouraging prospects, several challenges must be addressed before these tools can be fully realized in clinical practice. The tendency of chatbots to mishandle complex clinical nuances or produce unsafe recommendations in difficult cases underscores the need for continuous human oversight. Concerns regarding algorithmic bias, privacy, and legal accountability also demand careful consideration. Ensuring compliance with regulations like GDPR and HIPAA, establishing transparent development practices, and clarifying liability for AI-driven guidance are all essential steps in fostering trust and safeguarding patient safety.

Even so, emerging data emphasize the constructive role AI can play. For example, models like ChatGPT-4 have achieved a satisfactory level of accuracy in diagnosing bloodstream infections and recommending empirical antibiotic therapies—approximately 64% in one study [20]. Another study highlighted ChatGPT’s ability to provide nuanced responses to theoretical and open-ended clinical queries, at times outperforming infectious disease residents in specific tasks such as antibiogram interpretation, though it struggled to adapt to newer treatment guidelines [19]. While AI tools like ChatGPT can offer valuable insights, they still occasionally fail to manage complex clinical scenarios without expert supervision [17]. Nonetheless, these initial successes underscore AI’s potential to enhance guideline adherence, streamline decision-making, and possibly shorten the time to effective treatment.

As the technology advances, ongoing refinement of algorithms, robust clinical validation, interdisciplinary collaboration, and vigilant regulatory oversight will be crucial. Such efforts can help AI-driven chatbots evolve into reliable adjunct tools that complement human expertise and strengthen the global response to AMR, transforming what might seem like a high-tech promise into a tangible, enduring asset in healthcare.

## 10. Future Perspectives

To fully realize the potential of AI-based chatbots in optimizing antibiotic therapy, several critical areas demand sustained attention. First and foremost, refining the underlying algorithms and validating them clinically are essential steps. This includes improving the chatbot’s situational awareness so that it can better interpret complex clinical scenarios. In parallel, robust multicenter clinical trials are needed to confirm both efficacy and safety, focusing on prescribing error rates, infection resolution times, and the progression of antimicrobial resistance. Such studies should compare chatbot-generated recommendations with those of infectious disease specialists, taking into account factors such as time to correct antibiotic selection, cost-effectiveness, and adherence to official guidelines. By clarifying these outcomes, AI solutions can move from mere pilot projects to well-established tools in patient care.

Equally important is addressing the ethical and legal landscape. Strong data privacy and security measures, aligned with regulations like GDPR and HIPAA, must be embedded from the outset. Clinicians and patients alike need transparent, explainable AI systems they can trust, and clear guidance on accountability for AI-assisted clinical decisions is urgently needed to establish confidence and reliability in these tools.

On a practical level, interdisciplinary collaboration should become the norm. Clinicians, AI developers, ethicists, and policymakers must work together to develop and refine these technologies. Education and training for healthcare professionals will be key to helping them leverage AI chatbots as supportive instruments rather than stand-alone replacements for human judgment.

Integrating chatbots into existing clinical workflows will also require thoughtful design. Seamless incorporation into electronic health records, coupled with user-friendly interfaces, can help clinicians across varying levels of technological proficiency engage with the tool effectively. Meanwhile, a global perspective is crucial. Tailoring AI solutions to resource-limited settings, enabling offline functionality, providing language localization, and ensuring diverse training datasets can help reduce health disparities and extend the benefits of AI chatbots to underserved populations.

Looking ahead, AI chatbots have the potential to significantly enhance antibiotic selection and clinical outcomes by integrating real-time guideline updates, personalized local-resistance data, and advanced decision-support tools. For instance, in pediatric settings—where age-specific dosing and resistance patterns differ from adults—chatbots could deliver tailored, guideline-based recommendations aimed at minimizing broad-spectrum antibiotic use and reducing adverse events. Similarly, in critical care units, AI models capable of rapidly analyzing patient comorbidities, microbiological data, and pharmacokinetic-pharmacodynamic factors could help prevent overtreatment and shorten the time to effective therapy. To achieve these gains, however, future refinements must address the current limitations uncovered in recent studies: AI chatbots will require ongoing access to robust, high-quality datasets, continuous oversight by specialists, and transparent mechanisms to adapt recommendations when evidence evolves. By bridging these technical and practical gaps, AI chatbots stand to become indispensable adjuncts to clinical teams, offering timely and context-aware insights that can optimize antibiotic use and ultimately improve patient outcomes.

Regulatory compliance and standardization form the final cornerstone of progress. Close collaboration with bodies like the FDA and EMA is needed to establish clear standards for the approval, monitoring, and ongoing assessment of AI-based medical devices. Continuous post-market surveillance will allow for rapid identification and resolution of any emerging issues, thus promoting a stable and credible environment for these innovations. Consortia formed by industry, academia, and regulatory agencies might further unify testing protocols, ensuring transparency and adaptability throughout a model’s lifecycle. Meanwhile, structured educational programs—workshops, online courses, interactive simulations—will equip clinicians to interpret AI-driven recommendations critically, fostering trust and thoughtful use.

Over time, iterative improvements in data governance, such as using federated learning to preserve privacy, and cautious pilot implementations in controlled clinical environments will guide the steady, informed introduction of AI chatbots into routine practice. By following these paths, the field can move beyond mere proof-of-concept, ultimately codifying best practices, influencing policy, and supporting global alliances that transform what might seem like a high-tech promise into a tangible, enduring reality in healthcare.

## Figures and Tables

**Table 1:** **Key Findings from Studies on AI Chatbots in Antibiotic Therapy**. This table presents an overview of selected studies evaluating AI chatbots in managing infectious diseases. It includes details on study design, infection types, primary outcomes, key limitations, and implications for clinical practice.

Study Reference (Author, Year)	Study Design and Setting	Infection Type	Primary Outcomes	Key Limitations	Implications
Maillard et al., 2023, [20]	Prospective cohort study, tertiary hospital	Bloodstream infections (BSIs)	64% adequate empirical therapies, 36% optimal definitive therapies	Inadequate source control in some cases, long treatment durations	Useful as a supplementary tool, requires oversight
De Vito et al., 2024, [19]	Comparative study, single center	Various bacterial infections (BSIs, pneumonia, etc.)	70% accuracy in theoretical questions, limitations in resistance mechanism recognition	Older antibiotic preferences, limited guideline alignment	Promising in education, unsuitable for complex decisions
Sarink et al., 2023, [18]	Retrospective analysis, tertiary hospital	Positive blood cultures	Mean accuracy 2.8/5, highest for blood cultures	Ambiguous recommendations, occasional factual inaccuracies	Cannot replace clinicians, serves as diagnostic aid
Howard et al., 2023, [17]	Qualitative exploratory research, single center	General antimicrobial advice	Recognized contraindications inconsistently; proposed harmful recommendations	Failures in situational awareness, inconsistent inference	Needs human supervision, risk of dangerous advice

## Data Availability

No new data were created or analyzed in this study. Data sharing is not applicable to this article.
